# Use of a hygroscopic heat-and-moisture exchanger during the rapid shallow breathing index

**DOI:** 10.1186/cc14704

**Published:** 2015-09-28

**Authors:** Rodrigo C Borges, Leda TY Silveira, Juliana B Fernandes, Natalia S Arco, Samira P Furtado, Alexandra S Colombo

**Affiliations:** 1 Hospital Universitário da Universidade de São Paulo, SP, Brasil

## Introduction

Heat-and-moisture exchangers (HMEs) have been increasingly used to heat and humidify the inspired gases in patients undergoing mechanical ventilation. However, little is known about its interference during measurement of the rapid shallow breathing index (RSBI).

## Objective

The objective of this study was to evaluate the effect of the use of a hygroscopic HME during the measurement of RSBI in patients under mechanical ventilation (MV).

## Methods

Randomized and controlled clinical study in patients admitted to the ICU. Inclusion criteria were patients of age ≥18 years, with MV for at least 24 hours through an orotracheal tube (OTT) with an internal diameter of 7.5-9.0 mm, who were in the process of MV weaning. Patients were randomly allocated into the HME group or the non-HME group by a nonrelated investigator. Before the RSBI, subjects received respiratory physical therapy (bronchial hygiene therapy and tracheal suctioning) and were ventilated at pressure support ventilation with PEEP = 5-8 cmH_2_O, pressure support = 7-12 cmH_2_O, FiO_2 _<40 %, aiming peripheral O_2 _saturation >90 %, tidal volume between 6 and 8 ml/kg and respiratory frequency <30 bpm for 30 minutes. Soon after, minute ventilation, dynamic compliance, airway resistance and respiratory frequency were registered by MV. The bed head was elevated at 45°, subjects ventilated with FiO_2 _= 100 % for 1 minute, then disconnected from the ventilator, and the RSBI was then measured with a spirometer connected to the patient's OTT. For the HME group, a hygroscopic HME (Bact-HME; Pharma Systems, Knivsta, Sweden) was placed between the spirometer and the patient's OTT. Other collected data were clinical history (comorbidities, diagnosis, motive of admission), antropometric data, SAPS III and Sepsis-related Organ Failure Assessment (SOFA) at the first 24 hours in the ICU, MV duration, ICU length of stay, presence of sepsis, use of corticoids, vasoactive drugs and dialysis days, and laboratorial examinations.

## Result

Twenty-six subjects were assessed: 14 in the HME group and 12 in the non-HME group. Groups were not different regarding clinical conditions (comorbidities, cause of intubation, SAPS III, SOFA, MV duration, MV parameters, respiratory parameters, dose of sedation up to the moment of the RSBI measurement). The non-HME group presented a higher vasoactive drug dose (*p *= 0.04). No statistical significance was found between the measured RSBI for the HME group and the non-HME group.

## Conclusion

Use of a hygroscopic HME filter does not interfere with RSBI measurement and could be an alternative to avoid spirometer contamination.

